# Statistical learning in children with a family risk of dyslexia

**DOI:** 10.1002/dys.1711

**Published:** 2022-03-14

**Authors:** Elise de Bree, Josje Verhagen

**Affiliations:** ^1^ Development and Education of Youth in Diverse Societies, Utrecht University Utrecht the Netherlands; ^2^ Amsterdam Center for Language and Communication, University of Amsterdam Amsterdam the Netherlands

**Keywords:** dyslexia, family risk, non‐adjacent dependency learning, serial reaction time task, statistical learning

## Abstract

The assumption that statistical learning is affected in dyslexia has generally been evaluated in children and adults with diagnosed dyslexia, not in pre‐literate children with a family risk (FR) of dyslexia. In this study, four‐to‐five‐year‐old FR children (*n* = 25) and No‐FR children (*n* = 33) completed tasks of emerging literacy (phoneme awareness and RAN). They also performed an online non‐adjacent dependency learning (NADL) task, based on the Serial Reaction Time (SRT) task paradigm. Children's accuracy (hits), signal sensitivity (*d*′) and reaction times were measured. The FR group performed marginally more poorly on phoneme awareness and significantly more poorly on RAN than the No‐FR group. Regarding NADL outcomes, the results were less straightforward: the data suggested successful statistical learning for both groups, as indicated by the hit and reaction time curves found. However, the FR group was less accurate and slower on the task than the No‐FR group. Furthermore, unlike the No‐FR group, performance in the FR group varied as a function of the specific stimulus presented. Taken together, these findings fail to show a robust difference in statistical learning between children with and without an FR of dyslexia at preschool age, in line with earlier work on older children and adults with dyslexia.

## 1 STATISTICAL LEARNING IN CHILDREN WITH A FAMILY RISK OF DYSLEXIA

Dyslexia is a disorder characterized by consistently poor word reading and spelling performances that cannot be accounted for by general learning difficulties, sensory or cognitive deficits, or inadequate teaching (Peterson & Pennington, 2015). In order to gain more understanding of this disorder, we assess whether statistical learning is a potential underlying difficulty of dyslexia. Statistical learning refers to the ability to rapidly and automatically extract regularities from the environment over time (Schapiro & Turk‐Browne, 2015). In this study, we compare online performance on a serial reaction time (SRT) task measuring non‐adjacent dependency learning in kindergarten children with and without a family‐risk (FR) of dyslexia.

Statistical learning could be affected in FR children for two reasons. First, the ability to read and spell depends, in part, on statistical learning (e.g., Arciuli, Monaghan, & Seva, 2010; Pacton, Perruchet, Fayol, & Cleeremans, 2001; Treiman, 2017). Specifically, learning to read and spell requires learning grapheme‐phoneme correspondences. This, in turn, requires the knowledge that a grapheme can be mapped to multiple phonemes and vice versa. The frequency of these different possible mappings provides probabilistic cues about the pronunciation of a grapheme within a word, depending on its neighbouring graphemes. Such probabilities are used by readers and spellers from a young age onwards (e.g., Cassar & Treiman, 1997; Pacton & Fayol, 2000) and used for constructing orthographic representations (Frost, Siegelman, Narkiss, & Afek, 2013), which is needed to become literate.

A second reason why statistical learning could be affected in children with (an FR of) dyslexia is related to the interpretation that statistical learning is a measure of procedural learning. A procedural learning deficit has been proposed to account for the broader deficits associated with dyslexia (Nicolson & Fawcett, 2007, 2011; Ullman & Pierpont, 2005), such as difficulties in motor control (Chaix et al., 2007), attention (McGrath et al., 2011), and oral language (McArthur, Hogben, Edwards, Heath, & Mengler, 2000). The procedural learning deficit hypothesis (Nicolson & Fawcett, 2007; Ullman & Pierpont, 2005) is grounded in the idea that (sensori‐)motor and language‐related tasks depend on the same brain regions as the procedural memory system, and that people with dyslexia have a deficit in this system. Following this line of reasoning, poorer performance for children with (an FR of) dyslexia could be expected on procedural learning tasks, including those assessing statistical learning.

Support for the connection between statistical learning and literacy stems from two strands of research. First, earlier studies have shown that statistical learning is associated with literacy abilities in typically developing children and adults (e.g., Apfelbaum, Hazeltine, & McMurray, 2013; Arciuli & Simpson, 2012; Chetail, 2017; Frost et al., 2013; Ise, Arnoldi, Bartling, & Schulte‐Körne, 2012; Pollo, Kessler, & Treiman, 2009) and children with dyslexia (e.g., van der Kleij, Groen, Segers, & Verhoeven, 2018). Second, children and adults with dyslexia have been reported to have lower statistical learning skills as compared to their non‐dyslexic peers (e.g., Lum, Ullman, & Conti‐Ramsden, 2013; Pavlidou, Kelly, & Williams, 2010; Vicari, Marotti, Menghini, Molinari, & Petrosini, 2003 for a meta‐analysis).

Despite these reports on poorer statistical learning in children and adults with dyslexia, findings are not consistent. For instance, relationships between statistical learning and literacy are not always attested (e.g., Schmalz, Moll, Mulatti, & Schulte‐Körne, 2018; van Witteloostuijn, Boersma, Wijnen, & Rispens, 2021). Furthermore, not all studies report poorer statistical learning in people with dyslexia (He & Tong, 2017; Kelly, Griffiths, & Frith, 2002; Staels & Vanden, 2017) or even report steeper learning curves, suggesting more successful statistical learning, for people with dyslexia (Bennett, Romano, Howard Jr., & Howard, 2008). A recent and comprehensive study by van Witteloostuijn, Boersma, Wijnen, and Rispens (2019), for example, found that children with dyslexia did not differ from typically developing children on statistical learning in an SRT task, a visual statistical learning task and a miniature language learning task. Despite looking at these three domains and looking at online and offline measures of learning, no group differences were attested. Recent systematic reviews and meta‐analyses (Schmalz, Altoè, & Mulatti, 2017; van Witteloostuijn, Boersma, Wijnen, & Rispens, 2017; West, Hulme, & Melby‐Lervåg, 2021) further indicate that group differences between populations with and without dyslexia are not consistent and sometimes subject to publication bias (Schmalz et al., 2017; van Witteloostuijn et al., 2017).

Earlier work on relationships between dyslexia and statistical learning has concentrated on adults and children diagnosed with dyslexia. Studies into pre‐literate children's statistical learning abilities are scarce. However, such studies are important, as they can determine whether statistical learning is a potential difficulty *preceding* a literacy deficit. When assessing statistical learning in children whose literacy instruction has commenced and whose disorder has been diagnosed, the cause and consequence of the literacy disorder cannot be teased apart. If a statistical learning deficit is related to dyslexia, then the statistical learning performance of a group of children with an FR is expected to be poorer than in a group of No‐FR children. Children with an FR of dyslexia show an increased risk of developing dyslexia (Snowling & Melby‐Lervåg, 2016). Despite the heterogeneity within the group, with only part of the FR group developing literacy difficulties, earlier work has shown that FR groups as a whole have poorer emergent literacy abilities than their No‐FR peers (Snowling & Melby‐Lervåg, 2016; van Viersen et al., 2018). Thus, if statistical learning is a correlate of dyslexia, FR children might also show a deficit in this domain.

The few studies that are available on statistical learning in FR populations have found mixed results. Capel (2018) reported results of sequential visuospatial and language learning tasks of 8‐month‐old infants with and without an FR of dyslexia. Whereas results on the sequential language learning task could not be interpreted, as no effects of learning were attested in either of the groups, the results on the visuospatial sequential learning tasks did not show significant differences between the FR and No‐FR groups.

In contrast, Kerkhoff, de Bree, de Klerk, and Wijnen (2013) did find differences between 18‐month‐old infants with and without an FR of dyslexia on a statistical learning task. In the non‐adjacent dependency learning (NADL) task used in this study, infants were exposed to strings with an *a*‐*X*‐*b* and *c*‐*X*‐*d* structure, in which *X* varied, but *a*, *b*, *c*, and *d* were kept constant. The occurrence of *b* thus depended on that of *a* and occurrence of *d* on *c*. Such non‐adjacent dependency relations surface in written languages (e.g., the pronunciation of grapheme “*a*” is dependent on a nonadjacent grapheme, as in *mane* vs. *man*). They also occur in spoken languages (e.g., the English progressive, in which “is” and “‐ing” are constant and the verb stem varies, as in “John is swimming”). In the experiment by Kerkhoff et al., exposure to an auditory speech stream of non‐adjacent dependency relations (training phase) was followed by a test phase in which both familiar strings, conforming to the sequences exposed to during the training (*a*‐*X*‐*b* and *c*‐*X*‐*d*) and novel strings, violating these sequences (*a*‐*X*‐*d* and *c*‐*X*‐*b*), were presented. The results showed that infants without an FR of dyslexia showed longer looking times to the violations, whereas FR infants did not show a difference in looking times between the strings that conformed to the rules and those that did not. This lack of discrimination in the FR infants was taken as support for a statistical learning deficit.

These findings by Kerkhoff et al. (2013) are interesting in light of the potential statistical learning difficulties in dyslexia. However, they are limited by the fact that (pre‐)literacy skills cannot be charted yet at such a young age, so it could not be assessed whether the FR group truly differed from the No‐FR group on literacy(−related) abilities. Furthermore, the behavioural experimental methods that can be used with infants for assessing statistical learning do not allow capturing the learning trajectory during the task: knowledge of the artificial language acquired during the training is assessed post‐hoc, in a separate test phase. In contrast, online tracking is a way to assess learning continuously. It can reveal insight into the dynamic nature of the learning process and thus focus not only on what is learned but also on how learning takes place (Siegelman, Bogaerts, Kronenfeld, & Frost, 2018). An analysis of online statistical learning can therefore provide insight into the learning curve, and reveal whether FR and No‐FR children show differences during learning. Finally, a possibility that cannot be excluded in a classical training‐test design is that poor discrimination ability in the test phase is due to participants being confused, or even “un‐learning” the pattern presented during the training, as a result of being exposed to violations to this pattern at test. Online statistical learning assessments that provide information on participants' performance during their encounter of the stimuli do not have this drawback.

A commonly used online task of statistical learning is the Serial Reaction Time task (SRT) task. In SRT tasks, participants respond to a fixed set of stimuli that together form a pattern that is not explicitly taught (Nissen & Bullemer, 1987). As participants become more sensitive to this pattern, they give faster and more accurate responses. In a typical SRT task, participants are asked to respond as fast as possible to a visual stimulus appearing in one of four locations on a screen. Unknown to participants, there is a pattern in the presentation of the stimuli. After a number of learning blocks, in which all stimuli presented to conform to a single rule or single set of rules, a final block is presented, in which the pattern is disrupted. If performance increases over the regular blocks, this can be taken to reflect both practice and learning of the pattern. If performance drops from the regular to the disruption block, this is taken to reflect learning of the pattern. For reaction times the opposite pattern is expected: if learning takes place, participants should show a decrease in reaction times across the regular blocks, followed by an increase from the final regular block to the disruption block.

## 2 THIS STUDY

In the present study, we compared the performance of kindergarten children with and without an FR of dyslexia on a statistical learning task, specifically, a non‐adjacent dependency learning task (NADL) with an SRT design that has been used successfully in previous research (Verhagen & de Bree, 2020). Although statistical learning has been assessed through SRT tasks in adults and children with dyslexia, no (NADL) SRT studies have yet looked at FR children this young. If statistical learning is related to dyslexia, difficulties are expected to surface in children preceding the actual literacy instruction and outcomes.

We tested the hypothesis that the FR group would show poorer performance on the NADL SRT task than the No‐FR group, evidenced by a less steep learning curve during the regular blocks of this task and a smaller drop in performance from the regular blocks to the final, disruption block. Note, however, that this prediction was tentative, as earlier research on statistical learning in school‐aged children and adults with dyslexia has shown mixed results. Similarly, studies with FR infants showed mixed results, with some research showing that NADL is poorer in FR toddlers. No earlier studies have looked at FR kindergartners yet.

We also compared children's outcomes on tasks of emergent literacy skills in the FR and no FR‐groups. Phonological awareness (PA) and rapid automatized naming (RAN) tasks are associated with later literacy outcomes (Melby‐Lervåg, Lyster, & Hulme, 2012) and have been shown to distinguish FR and No‐FR children (Snowling & Melby‐Lervåg, 2016, with effect sizes [Cohen's *d*] of −.56 for PA and −.61 for RAN). We expected poorer performance on these tasks in the FR‐group than in the No‐FR group.

## 3 METHOD

### 3.1 Participants

Participants were 58 Dutch monolingual four‐ and five‐year‐old children (*M*_age_ = 64.2 months, *SD* = 5.8 months, range 51–71 months). Of these, 33 children had no increased family risk of dyslexia (No‐FR group) (*M*_age_ = 63.6 months, *SD* = 5.9, range 51–71, 20 girls), and 25 children had an increased family risk (FR group) (*M*_age_ = 64.9 months, *SD* = 5.6, range 53–71, 13 girls). Specifically, children in the FR group had at least one parent who had reported a diagnosis of dyslexia and/or a history of severe reading/spelling problems in a questionnaire. Age and gender did not differ between the groups (*t*(56) = 0.758 *p* = .396 for age; *χ* ^2^(1) = 0.430, *p* = .512 for gender). Six additional children were tested but excluded (*n* = 4 FR) due to extremely low scores in the NADL SRT task (i.e., no correct responses in one or more blocks) (*n* = 2), unwillingness to complete this task (*n* = 1), or experiment error (*n* = 3). Children were recruited through elementary schools (*n* = 36) and through the Babylabs of Utrecht University and Leiden University (*n* = 22). Written informed consent was obtained from children's parents.

## 4 MATERIALS

### 4.1 Literacy‐related measures

#### 4.1.1 Phoneme awareness

Phoneme awareness (PA) was measured using a word‐initial phoneme task (de Jong, 2007). In this task, children saw four pictures, one of which was the target picture (e.g., ball). Children were then asked to indicate which of the other three pictures depicted an object that started with the same sound as the target picture (e.g., bear, doll, phone). For the first four items, the experimenter named the target picture (ball) and provided the first sound of its label (/b/). Next, the assessor labelled the three other pictures (bear, doll, phone) and asked the child to specify which picture was described with a word that started with the same first sound as that of the target picture. From item five onwards, the first sound of the target picture was no longer labelled and the child had to indicate straight away which of the three other pictures displayed a word that started with the same sound. There were two practice items and 12 test items. The correct score was tallied for each item. Reliability of the task was high in the study by Mulder, Verhagen, van der Ven, Slot, and Leseman (2017) in a sample of 497 Dutch five‐year‐olds (*α* = .84) and sufficient in the current data (*α* = .68).

#### 4.1.2 Rapid automatized naming

Rapid automatized naming (RAN) colours and symbols were assessed with subtests of the test for Continuous Naming and Word Reading (*CB&WL*; van den Bos & Lutje Spelberg, 2007). Children were presented with five columns of ten items each and asked to name all 50 items as quickly and accurately as possible. The score consisted of the number of items named correctly per second. Reported split‐half reliability for 6‐year‐olds is .80 for colours and .73 for pictures (van den Bos & Lutje Spelberg, 2007) for children slightly older than our participants.

### 4.2 Statistical learning experiment

Statistical learning was measured through a non‐adjacent dependency learning (NADL) task, using a reaction‐timed task based on the SRT task paradigm (see also Verhagen & de Bree, 2020). Typical SRT tasks require participants to press a button as quickly as possible upon the appearance of a visual specific stimulus, sometimes paired with an auditory stimulus (Hunt & Aslin, 2001; Lum et al., 2013; Vicari et al., 2003).

In the current NADL SRT task, children were presented with auditory speech strings that consisted of three components, or triplets (see Table 1). Two languages were created in which the pattern of the triplets was *a*‐*X*‐*b* and *c*‐*X*‐*d* (Language 1) or *a*‐*X*‐*d* and *c*‐*X*‐*b* (Language 2). In these triplets, *a*, *b*, *c*, and *d* were always the same, but 18 different targets could fill the *X* slot. In Language 1, the triplets were *a*‐*X*‐*b* (i.e., *rak‐X‐toef*) and *c*‐*X*‐*d* (*sot‐X‐lut*). In Language 2, the triplets were *a*‐*X*‐*d* (i.e., *rak‐X‐lut*) and *c*‐*X*‐*b* (i.e., *sot‐X‐toef*).

**TABLE 1:** Descriptive statistics of literacy‐related skills in the No‐FR and FR Groups

	No‐FR (*n* = 33)		FR (*n* = 24)		Cohen's *d*
	*M*	(*SD*)	*M*	(*SD*)	
PA (sum correct)	7.30	(1.69)	6.29	(2.20)	.52
RAN Colours (nr. colours per sec)	1.50	(0.43)	1.24	(0.37)	.65
RAN pictures (nr. pictures per sec)	1.52	(0.42)	1.18	(0.33)	.90

Stimuli and triplets were the same as in de Bree, Verhagen, Kerkhoff, Doedens, and Unsworth (2017) and Verhagen and de Bree (2020). They were based on the English stimuli used in Gómez (2002) and Gómez and Maye (2005) but adapted for Dutch phonemes and phonotactics. The following 18 two‐syllable pseudowords were used as X items: *banip*, *bensim*, *domo*, *fidan*, *hiftam*, *kasi*, *kengel*, *mofig*, *naspoe*, *noeba*, *plizet*, *poemer*, *rogges*, *snigger*, *sulep*, *vami*, *wadim*, *wiffel*.

There were four blocks in the task. In the first three blocks, all stimuli were regular, containing only triplets conforming to the pattern. In Language 1, these were *a*‐*X*‐*b*, *c*‐*X*‐*d* (*rak*‐X‐*toef*, *sot*‐X‐*lut*). In Language 2, these were *a*‐*X*‐*d* and *c*‐*X*‐*b* (*rak*‐X‐*lut*, *sot*‐X‐*toef*). In the fourth, disruption block, the pattern was abandoned. Instead, in this block, six triplets were presented in which the final elements *lut* and *toef* were combined with their incorrect counterpart (e.g., *rak‐X‐toef* for the language that contained *sot‐X‐toef*). An additional 12 were paired with other initial elements that had not been presented during the previous blocks (e.g., *jik‐X‐toef*, *tep‐X‐toef*, 6 with *jik* and 6 with *tep*). For an overview of all stimuli, see Appendix A.

Although one of the non‐adjacent elements, *toef*, is an existing word in Dutch (meaning “dot”, as in a dot of whipped cream), we included it in our stimuli to use exactly the same stimuli as earlier work on statistical learning in Dutch children (De Bree et al., 2017; Verhagen & de Bree, 2020) and adults (Grama, Kerkhoff, & Wijnen, 2016). *Toef* is a highly infrequent word, occurring only 0.05 in every million (SUBTLEX, Keuleers, Brysbaert, & New, 2010), and probably even less so in child‐directed speech. The stimuli were based on the speech of a female native speaker of Dutch, who used a high‐pitched friendly voice. Triplets had been constructed by cross‐splicing the pseudo‐words from this pre‐recorded speech, to ensure uniformity of the stimuli and avoid co‐articulation effects (see de Bree et al., 2017 for details).

**FIGURE 1:**
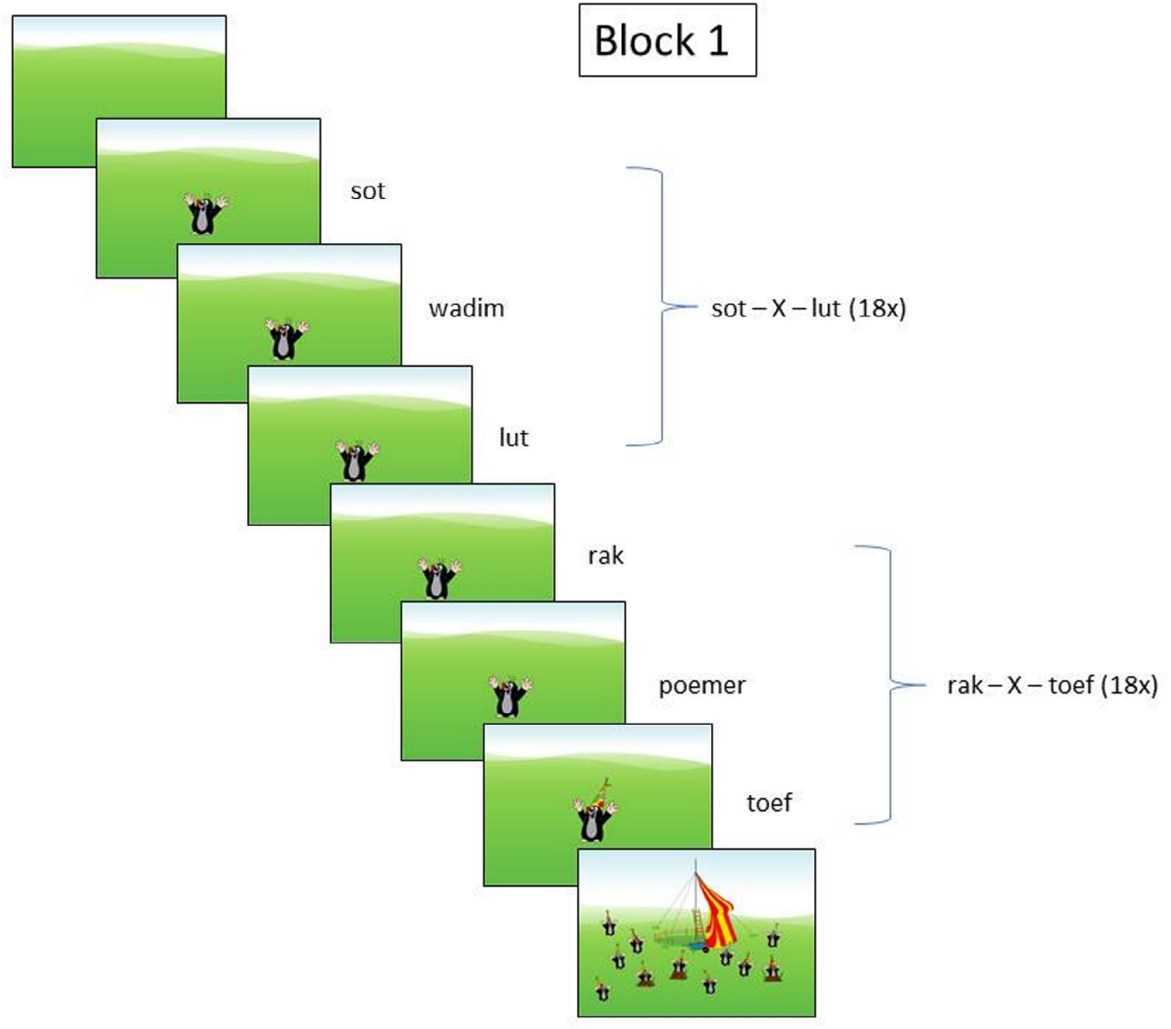
Illustration of block 1 in the Non‐Adjacent Dependency Learning SRT Experiment. In the experiment, triplets were presented in randomized order, and a mole with a party hat was shown when children pushed the button upon identifying a correct target

Triplets were presented to the children over headphones with a 250‐ms interval in between the elements in a triplet and a 750‐ms interval between triplets. At the start of the task, the children were told that they would see pictures of moles on the laptop screen and simultaneously hear the name of each mole. As all moles looked exactly the same (see Figure 1 below), they had to listen carefully to the names of the moles. The children were then told that there was going to be a party for moles and that their task was to “invite” to this party as many moles as they could, but only moles that were called *lut* (or *toef* in the counterbalanced version) should be invited. Children could invite these moles if they pressed a button on a push‐button box as quickly as possible when they heard a mole called *lut* (or *toef* in the other language). Children were also told that they did not need to wait for the mole called *lut* (or *toef*) to appear, but could also press the button when they thought the mole would appear. This procedure allowed for a comparison of accuracy and RT of children's responses across blocks.

Button presses were recorded from the offset of the X‐element to 750 ms after the offset of the final word of the triplet, (i.e., the name of the mole: *lut* or *toef*). If children pressed the button correctly, that is, if the triplet indeed contained the target word (a so‐called “hit”), the (“target”) mole got a party hat, showing children's correct response. If they did not press the button despite the presentation of the target, no feedback was given. If children did press the button when the target word was not present (a “false alarm” response), no feedback was provided either. This approach of positive feedback to correct answers only was used because previous piloting had established that children understood the task and were motivated to perform well when given positive feedback only. Thus, negative feedback was not considered necessary.

After each block, children were presented with a big colourful picture of all the moles they had “collected” during the immediately preceding block. Together with the experimenter, they counted the number of moles they had collected. They were provided with positive feedback irrespective of whether they had performed well on the preceding block(s), to keep them motivated for the next block.

The four test blocks (three regular blocks and one disruption block) were preceded by two practice blocks, in order to familiarize children with the task. In the first block, children were familiarized with the procedure of pressing the button *only* when the target word was played (e.g., *lut* or *toef*). In this practice block, 28 singlet items were presented, of which four were target words. In the second practice block, children were instructed to press the button as quickly as possible upon hearing the target: 28 singlets were presented, with four items being targets.

The first three, regular, test blocks all consisted of 18 triplets with a target word and 18 triplets of the other dependency (without a target word): the maximum number of target words per block was thus 18.^1^ Counterbalanced across experiment versions were the specific target word that children had to press the button for (i.e., *toef* vs. *lut*) as well as the language that children were presented with (Language 1: *rak‐X‐toef* and *sot‐X‐lut* vs. Language 2: *rak‐X‐lut* and *sot‐X‐toef*), resulting in four experiment versions. The fourth, disruption, block also contained 18 targets, but these were combined with first elements in triplets that did not conform to the non‐adjacent dependencies and thus were not predictable.

The experiment was programmed in the Zep software (http://beexy.org/zep/) and administered on a laptop computer. A button box was used to record children's responses. For each trial, accuracy (hit/false alarm) and response time (in the case of a button press) were recorded.

Split‐half reliability of the statistical learning task was computed using a Spearman–Brown corrected Pearson correlation (see also Siegelman, Bogaerts, & Frost, 2017; van Witteloostuijn et al., 2021). Specifically, split‐half reliability was calculated for each child as the correlation between the difference in RT between the random block (block 4) and the preceding regular block (block 3) in *even* versus *odd* trials. A split‐half reliability of *r* = .86 was found, indicating good reliability for the task that even exceeds the psychometric standard of *r* = .80 proposed in earlier work (Nunnally & Bernstein, 1994; Streiner, 2003).

### 4.3 Procedure

Children were tested individually by a research assistant in a quiet room at their schools or in the Babylabs of Utrecht University and Leiden University. Task order was fixed: the statistical learning experiment was followed by the RAN, and PA tasks, respectively. The test session lasted about 45 minutes. Children were awarded a small gift after participation. The research was conducted in accordance with ethical standards as well as The Netherlands Code of Conduct for Scientific Practice.

### 4.4 Variables and analyses

In order to test our hypotheses, linear mixed‐effects regression analyses were run, using R version 3.4.1 (R Core Team, 2017) and the *lme4* package (Bates, Maechler, Bolker, & Walker, 2015). Regarding our first question, linear mixed‐effects models were run on children's scores on the phoneme awareness and RAN tasks as the dependent variables, with “group” (FR vs. No‐FR) as a fixed effect. The model for phoneme awareness had random intercepts for subjects and items, as well as a by‐item random slope for group. The model for RAN had random intercepts for subjects, but not for items, since scores in these tasks involved one data point per child (number of correctly named symbols per second).

Regarding our second question addressing potential group differences between the FR and No‐FR group in NADL SRT performance, linear mixed‐effects regressions were run on children's task scores with the fixed‐effect factors “group” (FR vs. No‐FR), “block” (1 to 4), and “target word” (*lut* vs. *toef*). Three linear mixed‐effect regressions were run, with three different dependent variables, following earlier work using this task (Verhagen & de Bree, 2020). Specifically, two variables were constructed that were based on children's response accuracy: hits and *d′*. The variable “hits” was a categorical variable that could take two values: “0” for responses for strings that contained a target word but for which there was no button press, “1” for responses to strings that contained a target word and for which there was a button press. *D′* is a statistic from signal detection theory (MacMillan & Creelman, 2005) which reflects the percentage of correct responses to targets (hits) relative to the percentage of incorrect responses to distractors (false alarms). By taking into account both hits and false alarms, *d′* controls for potential response bias, as with children pressing the button in response to each stimulus. *D′* is typically calculated with the following formula: *d′* = *Z*(hit rate) − *Z*(false alarm rate) (MacMillan & Creelman, 2005). A higher *d′* signals more accurate signal detection than a low score. We applied a correction to handle zero scores, for which *d′* cannot be calculated otherwise (Hautus, 1995; Stanislaw & Todorov, 1999). Specifically, we added a score of 0.5 to children's number of hits and children's number of false alarms and 1 to the total number of targets/non‐targets such that the formula for *d*′ was as follows: *d′* = *Z* (number of hits +0.5/total targets presented +1) − *Z* (number of false alarms + 0.5/total non‐targets presented + 1). Since *d′* is based on hit and false alarm rates, it was calculated at the block level (i.e., mean *d′* scores per block), rather than at the item level, unlike children's hit responses.

The third dependent variable was RT‐based: residual RTs for children's hit responses were calculated by subtracting the duration of the target word from the total reaction time for each hit. A final RT‐based measure—anticipations—was also computed (following Verhagen & de Bree, 2020), which reflected the frequency with which children actually pressed the button prior to hearing the target over blocks. However, anticipations were very infrequent overall: the FR and No‐FR groups made a total of 8 and 18 anticipations, respectively. Therefore, they were not analyzed in the present paper. Whereas three different dependent variables (hits, *d′* and reaction times) may seem many, we choose to include these because they reflected different aspects of performance: accuracy was reflected through hits and *d*′; response speed through reaction times. As for accuracy, two measures were included because our primary and most simple measure (hits) had the drawback that it did not take into account false alarm rates. In sum, we felt that each variable was informative for a different reason, and thus needed to be included in order to compare the two groups' performance on the task.

The mixed‐effects model for hits had a binary dependent variable and thus involved a generalized mixed‐effects model. The mixed‐effects models for *d′* and reaction times had continuous dependent variables and thus were linear models. In the models for hits and *d′*, random intercepts for subjects were included; in the model for reaction times, random intercepts for subjects and items (i.e., X‐elements) were included, to obtain the maximal random effect structure supported by the data. In all analyses, sum‐to‐zero contrast coding was applied, with contrasts set for group (FR: −1/2, No‐FR: +1/2), target word (*lut*: −1/2, *toef*: +1/2), and block. For block, contrasts were set such that performance was compared between block 1 and block 2 (block 1: −1/2, block 2: +1/2), between blocks 1 and 2 versus 3 (1:‐1/3, 2: −1/3, 3:2/3), and between blocks 3 and 4 (block 3: −1/2, block 4: +1/2). Note that, in particular, the last comparison (between block 3 and block 4) is crucial, as this comparison allows to assess whether learning is disrupted, and is typically used as a measure of learning in SRT‐studies (Lum et al., 2013). Language (Language 1 or Language 2) was not included in the final models, as it did not yield an effect. All scripts and data files can be accessed via the OSF database (see: https://osf.io/5qfgn/?view_only=9a3485184f244492a4a13585ce0d646a).

## 5 RESULTS

### 5.1 Literacy‐related skills in the No‐FR and FR groups

Scores on the literacy‐related tasks were available for all No‐FR children and all but one FR child. Descriptive statistics are presented in Table 1.

A generalized linear mixed‐effects model on children's PA scores with group as the fixed‐effect factor showed a trend towards an effect of group (*β* = .833, *SE* = 0.444, *t* = 1.878, *p* = .060), indicating that the FR children tended to perform more poorly on this task than the No‐FR children. For RAN, a linear mixed‐effects model showed a main effect of group (*β* = .284, *SE* = 0.099, *t* = 2.854, *p* = .006), but no effect of item type, that is, colours or symbols, (*β* = −.018, *SE* = 0.039, *t* = −0.472, *p* = .639) or interaction between group and item type (*β* = .083, SE = 0.077, *t* = 1.067, *p* = .290). These results indicated that the FR group named significantly fewer colours and pictures per second than the No‐FR group.

### 5.2 NADL SRT performance in the No‐FR and FR groups

**FIGURE 2:**
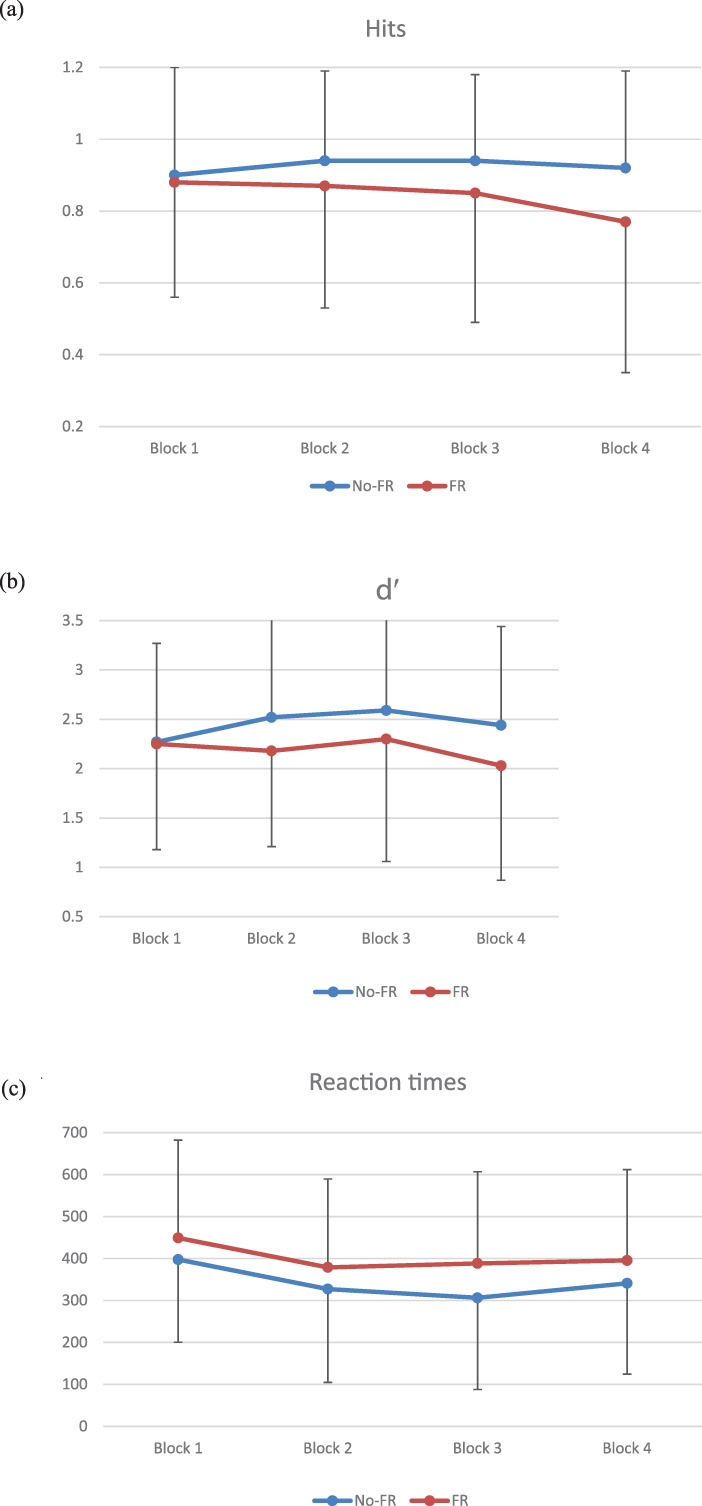
Mean results per block in the NADL SRT‐based task (and error bars) for the No‐FR and FR groups separately: (a) hit responses, (b) *d′*, (c) residualized reaction times for hits

Table 2 and Figure 2 contain mean scores and standard deviations per block for the two groups separately.

**TABLE 2:** Descriptive statistics per block of the NADL SRT‐Task for the No‐FR and FR Groups

	No‐FR (*n* = 33)		FR (*n* = 25)	
	*M*	(*SD*)	*M*	(*SD*)
Accuracy‐based variables				
Hits (mean probabilities)				
Block 1	0.90	(0.30)	0.88	(0.32)
Block 2	0.94	(0.25)	0.87	(0.34)
Block 3	0.94	(0.24)	0.85	(0.36)
Block 4	0.92	(0.27)	0.77	(0.42)
*d′* (mean *d*′)				
Block 1	2.27	(0.91)	2.25	(1.07)
Block 2	2.52	(1.00)	2.18	(0.97)
Block 3	2.59	(1.02)	2.30	(1.24)
Block 4	2.44	(1.12)	2.03	(1.16)
Reaction‐time based variable				
Residualized RTs to hits				
Block 1	397.64	(233.13)	449.15	(197.18)
Block 2	327.17	(210.59)	378.87	(222.04)
Block 3	306.35	(218.61)	388.34	(218.61)
Block 4	340.95	(216.32)	395.45	(216.32)

Visual inspection of these data shows typical SRT curves for hits, *d′* and reaction times for both groups: there is an increase in hits and *d*′ between blocks 1–2 and 2–3 and a decrease in reaction times between these blocks. In addition, there is a decrease in hits and *d*′ between blocks 3 and 4 and an increase in reaction times. The curves for the FR group suggest lower performance overall (fewer hits, longer reaction times) and are somewhat less clear overall, as they show a less steep increase in hits between the regular blocks 1, 2, and 3.

A generalized linear mixed‐effects model with hits as the dependent variable showed three main effects. First, there was a main effect of the group, which indicated that the FR‐group had fewer hits overall than the No‐FR group (*β* = 0.766, SE = 0.298, *z* = 2.570, *p* = .010). Second, a main effect of target word signalled that children were more likely to respond more accurately in the experiment versions with *lut* as the target word than in the experiment versions with *toef* as the target word (*β* = −.707, *SE* = 0.298, *z* = −2.369, *p* = .018). Third, a main effect of block showed that there was a significant decrease in hits between blocks 3 and 4 (*β* = −.408, *SE* = 0.192, *z* = −2.126, *p* = .033), but not between the first three regular blocks (all *p*s > .1). The model also rendered two significant two‐way‐interactions between block and group, which indicated that there was a steeper increase in hits for the No‐FR than FR group from block 1 to block 2 (*β* = .686, *SE* = 0.309, *z* = 2.222, *p* = .026) and from blocks 1 and 2 to block 3 (*β* = .956, *SE* = 0.343, *z* = 2.785, *p* = .005). A further, significant two‐way interaction between blocks 3 and 4 and target word (*β* = .933, *SE* = 0.384, *z* = 2.430, *p* = .015) indicated that the decrease in hits from block 3 to block 4 was stronger for the experiment versions with *lut* than the experiment versions with *toef*. Finally, there were two significant three‐way interactions. First, an interaction effect between group, blocks 1 and 2 versus block 3, and target word (*β* = −2.221, *SE* = 0.686, *z* = −3.238, *p* = .001) signalled that the FR group showed an increase in the number of hits from blocks 1 and 2 to block 3 in the experiment versions with *lut* but not with *toef* as a target word, whereas the No‐FR group showed this increase in hits with both target words. Second, and similar to this, an interaction between group, blocks 3 and 4, and target word (*β* = −2.486, *SE* = 0.767, *z* = −3.223, *p* = .001) indicated that the FR children showed a drop in performance from block 3 to block 4 with *lut* but not *toef*, whereas the No‐FR children did not show a difference in drop in performance between the target words. There were no other main effects or significant interactions in this model. For the results of the full model, see Table S1; for the descriptive results for versions with *lut* and *toef* separately, see Tables S1, S2 and S3.

A linear mixed‐effects regression model on *d′* scores with group, block and target word as its fixed‐effect factors showed no main effects. There was only a significant three‐way interaction between group, blocks 3 and 4, and target word. In line with the above results above for hit responses, the FR group showed a drop in performance from block 3 to block 4 for the experiment version in which *lut* was the target word but not in the version in which *toef* was the target word. The No‐FR group did not show this difference depending on target word (*β* = −1.504, *SE* = 0.576, *t* = −2.611, *p* = .010). There were no other main effects or interaction effects.

As for children's reaction times, a main effect of group indicated that the FR group responded more slowly overall than the No‐FR group (*β* = −72.545, *SE* = 30.427, *t* = −2.384, *p* = .021). Furthermore, the results showed a significant decrease in reaction times between blocks 1 and 2 (*β* = −73.803, *SE* = 9.039, *t* = −8.165, *p* < .001) and from blocks 1 and 2 to block 3 (*β* = −56.922, *SE* = 9.815, *t* = −5.779, *p* < .001), as well as a significant increase in reaction times from block 3 to block 4 (*β* = 25.815, *SE* = 11.777, *t* = −2.192, *p* = .029), irrespective of group. The other main effects and interactions were not significant (see Table S2).

## 6 DISCUSSION

This study assessed statistical learning in young children with a family risk (FR) of dyslexia. Specifically, two groups of four‐ and five‐year‐old children who either had an increased FR (FR group) or no increased FR (No‐FR group) performed a non‐adjacent dependency learning (NADL) task based on the serial reaction‐timed (SRT) task paradigm. Children also performed two tasks of emergent literacy: phoneme awareness (PA) and rapid automatized naming (RAN). Our results showed that the FR group obtained significantly lower scores on RAN and marginally significant scores on phoneme awareness than the No‐FR group. The effect sizes (Cohen's *d* ranging between .56 and .90) resembled those reported in a meta‐analysis on emergent literacy tasks in FR and No‐FR children (Snowling & Melby‐Lervåg, 2016). The NADL SRT‐based measure of statistical learning, which had good split‐half reliability, also showed some differences between the groups, but these were not clear‐cut.

Overall (FR and No‐FR group together), there were indications that statistical learning took place, as there was a decrease of reaction times during the regular (learning) blocks and an increase in the final (random) block. Similarly, there was a decrease in correct scores (number of hits) from the last regular block to the random block. Findings on the No‐FR group resembled those of a previously reported sample of monolingual children (Verhagen & de Bree, 2020) and were indicative of learning: there was an increase in performance over the regular blocks, followed by a decrease in the disruption block. There also was a decrease of signal sensitivity (*d*′) from the last regular block to the disruption block, but these differences turned out not to be significant.

The FR group differed from the No‐FR group, as the FR group obtained significantly fewer hits (button presses for strings that contained a target) than the No‐FR group overall, and was generally slower than the No‐FR group (higher RTs). As for responses over time (blocks), the results showed a less steep increase in hits over the regular blocks for the FR group as compared to the No‐FR group. Importantly, the performance of the FR group was affected by the stimuli used in the NADL SRT: the drop in performance from the three regular blocks to the random block was stronger for the experiment versions in which *lut* rather than *toef* was the target word. Similarly, for *d*′, a decrease in sensitivity from the last regular block to the random block was stronger for the experiment with *lut* than *toef* in the FR‐group, while there was no such difference in the No‐FR group.

The finding that the performance of the FR group was influenced by the specific target word in the non‐adjacent dependency presented is unexpected and difficult to interpret. A possible explanation is that there were differences in acoustic properties that led the FR group to respond differently to each nonword. However, this did not turn out to be the case: *lut* and *toef* had an equal duration (*toef*: 50 milliseconds, *lut*: 0.54 milliseconds), the same pitch movement (both with a minimum of approximately 220 Hz and a maximum at approximately 390 Hz), the same intensity (both an average of approximately 60 dB and a maximum of 65 dB), and the same intonation. Hence, the acoustic properties of the nonwords are unlikely to have played a role.

A second possible explanation for the effect of target word we found is that *toef* is an existing word in Dutch. Siegelman, Bogaerts, Elazar, Arciuli, and Frost (2018) found that previously assimilated verbal knowledge influenced statistical learning. However, as noted earlier, *toef* is a highly infrequent word in Dutch adult speech, and possibly even more so in child‐directed speech, decreasing the chances that children's pre‐existing verbal knowledge affected the outcomes on non‐adjacent dependency learning in the current experiment. Furthermore, it is difficult to see why the difference in response to *toef* and *lut* would only surface for the FR group.

A final, tentative interpretation is that the phonotactic probability of the target words affected the processing of the stimuli. The phonotactic probability of *lut* (−7.976) is higher (more frequent in Dutch) than that of *toef* (−12.067), as indicated by their mean log frequencies taken from the corpus of spoken Dutch (CGN, Oostdijk, 2000), and extracted with the software Phonotactools (Adriaans, 2006). Previous research has found that children with reading impairment have more difficulty in repeating non‐words with low compared to high phonotactic probability (Rispens, Baker, & Duinmeijer, 2015). Even though no repetition was elicited from the child in the current task, the same difficulty might affect word processing in FR children.

Our findings resemble those of Lammertink and colleagues in a similar non‐adjacent dependency task. They found that seven‐year‐old typically developing children responded faster when the target was *lut* than when the other target (*mip*, in their case) was used (Lammertink, van Witteloostuijn, Boersma, Wijnen, & Rispens, 2019). In a subsequent study, they found that this difference in response time between *lut* and *mip* was larger for nine‐year‐old children with developmental language disorder than for typically developing children (Lammertink, Boersma, Wijnen, & Rispens, 2020). Nevertheless, in contrast to our study, in which learning patterns of the FR group were different for the target words (*lut* and *toef*), this was not the case in the studies by Lammertink and colleagues. Effects of different versions of statistical learning experiments presented to participants might deserve further attention. Generally, “version” is only entered in statistical testing when a significant difference between different versions has first been established. We entered “version” as a fixed factor to all models and in interaction with the other predictors, to be able to see whether it influenced children's performance in some way or another. Based on our results as well as those by Lammertink et al. (2019), it seems that differences can arise on the basis of stimuli used, perhaps in particular in FR children and children with language disorders, which makes it worthwhile to check for such effects in any future statistical learning studies with young (FR) children.

Another important finding is that, despite some indications in our data that the FR children picked up the pattern less successfully than the No‐FR group, there were no pronounced group differences in NADL SRT performance. One possible explanation for this null effect is that many children in the FR group we assessed actually were not the ones who would develop the literacy disorder. This seems unlikely, however, since – at least at the group level – the FR children performed significantly more poorly on the emergent literacy measures than their No‐FR counterparts, with the effect sizes on PA and RAN closely resembling those reported in the meta‐analysis of Snowling and Melby‐Lervåg (2016).

A possible interpretation of our mixed pattern of results that cannot be excluded, then, is that statistical learning is only related to dyslexia and not to family risk. As our sample of children had not started literacy instruction yet and dyslexia can only be established by taking severity and persistence of the literacy difficulties into account, we do not know whether performance on statistical learning is related to the literacy deficit, to the family risk or neither. Specifically, we cannot determine whether only the subset of the FR‐children with actual dyslexia will be the ones who show poorer statistical learning, whether there is a stepwise pattern of FR + dyslexia < FR‐no dyslexia < control group, or no difference at all. Work by Moll, Loff, and Snowling (2013) has demonstrated that there is a stepwise pattern in children's phoneme awareness and nonword repetition (i.e., FR + dyslexia < FR‐no dyslexia < control group), indicating that these phonological skills are related both to family risk as well as actual literacy outcomes. Future work could establish whether similar group patterns hold for statistical learning.

The fact that our samples of FR and No‐FR children were not large might also have prevented significant differences from surfacing. Although our participant groups were similar in size to those in Kerkhoff et al. (2013), who did find group differences between FR and No‐FR toddlers, the tasks cannot be easily compared across studies. For the toddlers in Kerkhoff et al, looking times to the auditorily presented stimuli were measured, whereas in the current kindergartners, both auditory and visual information had to be processed and responses required button presses. Perhaps the increased task demands of our NADL SRT‐task as compared to a looking task as used in Kerkhoff et al. (2013) required larger samples for effects of FR on statistical learning to show up. However, research on older children has indicated that larger samples do not necessarily yield group differences. In their systematic review, Schmalz et al. (2018) found, for example, that the two larger SRT studies (>100 participants) were the ones in which no group differences surfaced between children with and without dyslexia. The same was true for the study by van Witteloostuijn et al. (2019), in which 8–10 year‐olds with (*n* = 50) and without dyslexia (*n* = 50) performed similarly on visual statistical learning and auditory nonadjacent dependency learning. Thus, while it is currently unclear whether our null results are, at least in part, due to the relatively small groups in our study, there are indications from the previous literature that null results may persist even with larger samples.

Our discussion so far has focused on the interpretation of our study being unable to find group differences on the NADL SRT task due to study limitations. An alternative interpretation of our findings is that statistical learning is not deficient in children with (an FR of) dyslexia. This interpretation would run counter to findings of the infant study of Kerkhoff et al. (2013) and to SRT findings reported in the meta‐analysis by Lum et al. (2013) on SRT performance in dyslexic and non‐dyslexic populations. It does, however, agree with more recent findings of meta‐analyses and systematic reviews. These do not find consistent and strong differences between populations with and without dyslexia on statistical learning in general (e.g., Schmalz et al., 2018; van Witteloostuijn et al., 2017; West et al., 2021), and for SRT in particular (Schmalz et al., 2018; West et al., 2021). Furthermore, they provide indications of publication bias playing a role in studies that do find differences between populations with and without dyslexia (for similar conclusions, see Schmalz et al., 2018; van Witteloostuijn et al., 2017).

In sum, our findings show some differences between the FR and No‐FR groups on a measure of non‐adjacent dependency statistical learning. However, these differences are not clear‐cut and seem to be modulated by the specific stimuli used. On the one hand, our findings could be taken to call for further research into (auditory and visual) statistical learning in this young age group, including larger sample sizes, and perhaps more sensitive (online) measures of statistical learning to see how FR children perform on statistical learning, also once they have become literate and clear distinctions between dyslexic and non‐dyslexic children can be made. On the other hand, our results fit the pattern of findings that statistical learning is not a (consistent) area of difficulty for children with dyslexia. Therefore, an alternative, and possibly more fruitful, direction for future research investigating statistical learning in dyslexic and FR children might be to look into more proximal and specific influences of statistical learning on the literacy process (Bogaerts, Siegelman, & Frost, 2021), such as the effects of word and grapheme frequency on children's abilities to read and spell (Pacton et al., 2001; Treiman, 2017).

## Supporting information


**Table S1.** Stimuli of the NADL SRT task
**Table S2.** Results of Linear Mixed‐Effect Models on NADL SRT Data with “Group”, “Block”, and “Target Word” as fixed‐effect predictor variables (effects with *p* < .05 are in bold)
**Table S3.** Descriptive statistics per block of the NADL SRT task for the No‐FR and FR Groups and the Two Target Words (Lut vs. Toef) Separately

## Data Availability

The data that support the findings of this study are available from the authors upon reasonable request.

## Endnote

^1^
One of the two experiment versions with *lut* as the target word contained an error in one of the versions such that 19 *lut* and 17 *toef* strings were accidentally presented in the regular blocks, instead of 18 instances of each type. This version was administered to 10 children (4 FR and 6 No‐FR) who thus were presented with 19 rather than 18 target words. To check whether this impacted our results, we repeated all the analyses without these 10 children included. The results of these analyses showed the same main effects and interactions, except that the effect of group in the models with hits and reaction times was no longer significant (*p* = .115 and *p* = .105, respectively), presumably due to reduced power. In the analyses reported in the paper “item” was included as a random‐effect factor to take into account the specific items that children were presented with. These analyses are based on the full sample of 58 children.
