# Supplementary material for: Statistical learning in children with a family risk of dyslexia
Source: Dyslexia. 2022 Mar 14;28(2):185–201. doi: 10.1002/dys.1711 (PMC9314089; doi:10.1002/dys.1711)
Supplement: Supplementary file 1 — Table S1. Stimuli of the NADL SRT task Table S2. Results of Linear Mixed‐Effect Models on NADL SRT Data with “Group”, “Block”, and “Target Word” as fixed‐effect predictor variables (effects with p < .05 are in bold) Table S3. Descriptive statistics per block of the NADL SRT task for the No‐FR and FR Groups and the Two Target Words (Lut vs. Toef) Separately [file DYS-28-185-s001.docx]

# Supplementary Files A

Table A1. *Stimuli of the Non-Adjacent Dependency Learning SRT Task*

| Experiment | Nr. of strings* | Language1 | |  | Language2 | |  |
| --- | --- | --- | --- | --- | --- | --- | --- |
|  |  | Non-adjacent dependencies | | | Non-adjacent dependencies | | |
| Block 1 | 2 x 18 | a-X_(1-18)_-b | c-X_(1-18)_-d | | a-X_(1-18)_-d | c-X_(1-18)_-b | |
|  |  | (rak X toef) | (sot X lut) | | (rak X lut) | (sot X toef) | |
| Block 2 | 2 x 18 | a-X_(1-18)_-b | c-X_(1-18)_-d | | a-X_(1-18)_-d | c-X_(1-18)_-b | |
|  |  | (rak X toef) | (sot X lut) | | (rak X lut) | (sot X toef) | |
| Block 3 | 2 x 18 | a-X_(1-18)_-b | c-X_(1-18)_-d | | a-X_(1-18)_-d | c-X_(1-18)_-b | |
|  |  | (rak X toef) | (sot X lut) | | (rak X lut) | (sot X toef) | |
| Block 4 | 2 x 18 | c-X_(1-6)_ –b | c-X_(1-18)_-d | | c-X_(1-6)_-d | c-X_(1-18)_-b | |
|  |  | (sot X toef)  e-X_(1-6)_-b  (tep X toef)  f-X_(1-6)_-b  (jik X toef) | (sot X lut) | | (sot X lut)  e-X_(1-6)_-d  (tep X lut)  f-X_(1-6)_-d  (jik X lut) | (sot X toef) | |
|  |  |  |  | |  |  | |

*Note.* The presented version per Language represents one of the two versions per Language; X_(subscript number)_ refers to the different X-items used. *See footnote 1 for a deviation in the number of strings presented in one of the experiment versions.

# Supplementary Files B

Table B1. *Results of Linear Mixed-Effect Models on NADL SRT Data with ‘Group’, ‘Block’, and ‘Target Word’ as Fixed-Effect Predictor Variables (effects with p < .05 are in bold)*

|  | *β* | SE | *t / z* | *p* |
| --- | --- | --- | --- | --- |
| *Hits* |  |  |  |  |
| Intercept | 2.530 | 0.151 | 16.721 | < .002 |
| Group | 0.766 | 0.298 | 2.570 | **.010** |
| Target word (*lut* vs. *toef*) | -0.707 | 0.298 | -2.369 | **.018** |
| Block (1 vs. 2) | 0.205 | 0.154 | 1.330 | .184 |
| Block (1/2 vs. 3) | 0.015 | 0.172 | 0.088 | .930 |
| Block (3 vs. 4) | -0.408 | 0.192 | -2.126 | **.033** |
| Group * Block (1 vs. 2) | 0.686 | 0.309 | 2.222 | **.026** |
| Group * Block (1/2 vs. 3) | 0.956 | 0.343 | 2.785 | **.005** |
| Group * Block (3 vs. 4) | 0.688 | 0.384 | 1.794 | .073 |
| Group * Target word | 0.257 | 0.596 | 0.430 | .667 |
| Block (1 vs. 2) * Target word | -0.539 | 0.309 | -1.746 | .081 |
| Block (1/2 vs. 3) * Target word | -0.177 | 0.343 | -0.514 | .607 |
| Block (3 vs. 4) * Target word | 0.933 | 0.384 | 2.431 | **.015** |
| Group * Block (1 vs. 2) * Target word | 0.995 | 0.617 | 1.611 | .107 |
| Group * Block (1/2 vs. 3) * Target word | -2.221 | 0.686 | -3.238 | **.001** |
| Group * Block (3 vs. 4) * Target word | -2.468 | 0.766 | -3.223 | **.001** |
| *d’* |  |  |  |  |
| Intercept | 2.408 | 0.115 | 20.871 | < .001 |
| Group | 0.229 | 0.231 | 0.994 | .324 |
| Target word (*lut* vs. *toef*) | 0.070 | 0.231 | 0.303 | .763 |
| Block (1 vs. 2) | 0.043 | 0.118 | 0.368 | .713 |
| Block (1/2 vs. 3) | 0.103 | 0.125 | 0.825 | .411 |
| Block (3 vs. 4) | -0.101 | 0.144 | -0.701 | .484 |
| Group * Block (1 vs. 2) | 0.396 | 0.235 | 1.684 | .094 |
| Group * Block (1/2 vs. 3) | 0.264 | 0.250 | 1.058 | .291 |
| Group * Block (3 vs. 4) | 0.220 | 0.288 | 0.764 | .446 |
| Group * Target word | -0.087 | 0.461 | -0.188 | .851 |
| Block (1 vs. 2) * Target word | -0.347 | 0.235 | -1.476 | .142 |
| Block (1/2 vs. 3) * Target word | -0.040 | 0.250 | -0.161 | .872 |
| Block (3 vs. 4) * Target word | 0.354 | 0.288 | 1.229 | .221 |
| Group * Block (1 vs. 2) * Target word | 0.327 | 0.470 | 0.694 | .488 |
| Group * Block (1/2 vs. 3) * Target word | -0.742 | 0.499 | -1.487 | .139 |
| Group * Block (3 vs. 4) * Target word | -1.505 | 0.576 | -2.611 | **.010** |
| *RTs* |  |  |  |  |
| Intercept | 385.120 | 15.974 | 24.109 | <. 001 |
| Group | -72.545 | 30.427 | -2.384 | **.021** |
| Target word (*lut* vs. *toef*) | 54.078 | 30.427 | 1.777 | .082 |
| Block (1 vs. 2) | -73.803 | 9.039 | -8.165 | **< .001** |
| Block (1/2 vs. 3) | -56.922 | 9.815 | -5.799 | **< .001** |
| Block (3 vs. 4) | -25.815 | 11.777 | -2.192 | **.029** |
| Group * Block (1 vs. 2) | -0.113 | 18.078 | -0.006 | .995 |
| Group * Block (1/2 vs. 3) | -12.930 | 19.263 | -0.671 | .502 |
| Group * Block (3 vs. 4) | 28.432 | 22.373 | 1.271 | .204 |
| Group * Target word | -109.452 | 60.853 | -1.799 | .078 |
| Block (1 vs. 2) * Target word | 0.256 | 18.077 | 0.014 | .989 |
| Block (1/2 vs. 3) * Target word | -6.450 | 19.268 | -0.335 | .738 |
| Block (3 vs. 4) * Target word | -30.597 | 22.376 | -1.367 | .172 |
| Group * Block (1 vs. 2) * Target word | 14.756 | 36.156 | 0.408 | .683 |
| Group * Block (1/2 vs. 3) * Target word | 17.984 | 38.525 | 0.467 | .641 |
| Group * Block (3 vs. 4) * Target word | 6.339 | 44.751 | 0.142 | .887 |

Table B2.

*Descriptive Statistics per Block of the NADL SRT-Task for the No-FR and FR Groups and the Two Target Words (Lut vs. Toef) Separately*

|  | No-FR (*n* = 33) | | | | FR (*n* = 25) | | | |
| --- | --- | --- | --- | --- | --- | --- | --- | --- |
|  | Lut | | Toef | | Lut | | Toef | |
|  | M | (SD) | M | (SD) | M | (SD) | M | (SD) |
| *Hits (mean probabilities)* |  |  |  |  |  |  |  |  |
| Block 1 | 0.91 | (0.29) | 0.89 | (0.32) | 0.90 | (0.30) | 0.86 | (0.35) |
| Block 2 | 0.95 | (0.23) | 0.93 | (0.26) | 0.92 | (0.26) | 0.77 | (0.42) |
| Block 3 | 0.97 | (0.16) | 0.90 | (0.29) | 0.91 | (0.29) | 0.74 | (0.44) |
| Block 4 | 0.95 | (0.21) | 0.90 | (0.31) | 0.78 | (0.41) | 0.76 | (0.43) |
| *D’ (mean d’)* |  |  |  |  |  |  |  |  |
| Block 1 | 2.08 | (0.93) | 2.45 | (0.88) | 2.25 | (1.13) | 2.26 | (1.04) |
| Block 2 | 2.42 | (1.15) | 2.60 | (0.86) | 2.35 | (1.09) | 1.92 | (0.70) |
| Block 3 | 2.53 | (1.27) | 2.65 | (0.77) | 2.49 | (1.24) | 2.01 | (1.25) |
| Block 4 | 2.45 | (1.27) | 2.44 | (1.01) | 1.85 | (1.25) | 2.30 | (1.00) |
| *Residualized RTs to hits* |  |  |  |  |  |  |  |  |
| Block 1 | 404.65 | (221.88) | 391.51 | (180.31) | 406.17 | (242.76) | 522.27 | (203.59) |
| Block 2 | 326.80 | (209.87) | 327.49 | (209.70) | 343.54 | (211.57) | 447.48 | (229.93) |
| Block 3 | 299.23 | (217.00) | 312.89 | (220.58) | 354.59 | (201.69) | 455.33 | (199.06) |
| Block 4 | 348.29 | (243.01) | 334.44 | (200.40) | 370.14 | (191.49) | 437.51 | (235.25) |
